# Medical Management of Polycystic Liver Disease: A Position Statement From the European Reference Network on Hepatological Diseases

**DOI:** 10.1111/liv.70451

**Published:** 2025-12-02

**Authors:** Sophia Heinrich, Andreia Margarida Carvalho de Matos, Jordi Colmenero, Ahmed M. Elsharkawy, Soňa Fraňková, Jan Halbritter, Anna Mrzljak, Roman‐Ulrich Müller, Rafaela Pereira, Pavel Strnad, Carmen A. J. Teemer, Jef Verbeek, Karolina M. Wronka, Frederik Nevens, Richard Taubert, Joost P. H. Drenth, the European Reference Network on Hepatological Liver Diseases (ERN RARE‐LIVER)

**Affiliations:** ^1^ Department of Gastroenterology, Hepatology, Infectious Diseases and Endocrinology Hannover Medical School Hannover Germany; ^2^ European Reference Network on Hepatological Diseases (ERN RARE‐LIVER) Hamburg Germany; ^3^ Unidade Local de Saúde de Coimbra, EPE, Unidade de Transplantacao Hepática de Adultos, Servico de Medicina Iinterna – Unidade Funcional de Doenca Hepática, Centro Hospitalar e Universitário de Coimbra Coimbra Portugal; ^4^ Liver Transplantation, Liver Unit, Hospital Clínic, Biomedical Research Institute August Pi i Sunyer (IDIBAPS), Biomedical Research Networking Center (CIBER) University of Barcelona Barcelona Spain; ^5^ HR Birmingham Biomedical Research Centre University Hospitals Birmingham NHS Foundation Trust, University of Birmingham Birmingham UK; ^6^ Liver Unit Queen Elizabeth Hospital Birmingham Birmingham UK; ^7^ Institut klinické a experimentální medicíny, IK+EM Praha Czech Republic; ^8^ Department of Nephrology and Medical Intensive Care Charité Universitätsmedizin Berlin Berlin Germany; ^9^ European Reference Network for Rare Kidney Diseases (ERKNet) Heidelberg Germany; ^10^ Liver Transplant Center, University Hospital Center Zagreb School of Medicine, University of Zagreb Zagreb Croatia; ^11^ Cluster of Excellence on Cellular Stress Responses in Aging‐Associated Diseases (CECAD) University of Cologne Cologne Germany; ^12^ Department II of Internal Medicine, University of Cologne Faculty of Medicine and University Hospital Cologne Cologne Germany; ^13^ Hospital Curry Cabral Lisboa Portugal; ^14^ Department of Medicine III University Hospital RWTH Aachen Aachen Germany; ^15^ University Hospitals KU Leuven Leuven Belgium; ^16^ Department of Hepatology, Transplantology and Internal Medicine Medical University of Warsaw Warsaw Poland; ^17^ Department of Gastroenterology and Hepatology Amsterdam University Medical Center Amsterdam the Netherlands

**Keywords:** hepatomegaly, liver volume, medical therapy, polycystic liver disease, somtatostatine analogues, symptom management

## Abstract

Polycystic liver disease (PLD) is a rare genetic disorder characterised by progressive liver enlargement due to multiple cysts. The main symptoms are liver volume‐related. Although randomised controlled trials have shown that somatostatin analogues (SSAs) reduce liver volume as well as symptoms, specific guidance on when and how to use SSAs in clinical practice is still lacking. A panel of 15 hepatologists and nephrologists developed practical guidance on SSA use, based on a systematic literature search, expert surveys, and clinical experience. This consensus was reached during a two‐day workshop by the European Reference Network on Hepatological Diseases, using 11 predefined key questions and an iterative Delphi process. PLD patients with liver volume‐related symptoms, diffuse disease, and significant hepatomegaly are eligible for SSA therapy, regardless of kidney function. Disease burden should be assessed with validated PROMs as well as liver volumetry before and during treatment. Symptom relief without liver growth is a valid treatment response. Therapy may continue until the natural course of disease slows down (e.g., with the onset of menopause). Trials report benefits of SSA therapy for up to 3–4 years. Early discontinuation may cause a rebound of liver volume. As SSA therapy remains off‐label, limited access and reimbursement hamper widespread use in Europe. We present state‐of‐the‐art guidance on the practical use of the only available medical therapy for severe PLD including eligibility, start and stop criteria and identifying research gaps. This consensus‐based guidance provides much‐needed practical recommendations for the use of somatostatin analogues in managing severe polycystic liver disease. By defining eligibility, treatment goals, and monitoring strategies, it supports more standardised, patient‐centered care across Europe.

**Summary**

- Patient selection: Consider SSA therapy in PLD patients with liver volume‐related symptoms and non‐localised disease.
- Baseline assessment: evaluate symptomatic burden with validated PROMs (PLD‐Q, POLCA), TLV (CT/MRI volumetry) and expected TLV trajectory.
- Treatment goal: aim primarily for relief of liver volume‐related symptoms
- Monitoring: Use PROMs and TLV to assess response
- Treatment duration: continue SSAs while benefits outweigh side effects; stop when treatment response is no longer achieved.

**Abbreviations**
ADPKD: autosomal dominant polycystic kidney disease
ADPLD: autosomal dominant polycystic liver disease
cAMP: cyclic adenosine monophosphate
ERN: European Reference Network
HRQoL: health‐related quality of life
hTLV: height adjusted total liver volume
LVrS: liver‐related symptoms
PLD: polycystic liver disease
PROM: patient‐reported outcome measurement
RCT: randomised controlled trials
SSA: somatostatine analogue
TLV: total liver volume

## 1 Introduction

Polycystic liver disease (PLD) arises as the common phenotype of two independent, inherited disorders: autosomal dominant polycystic liver disease (ADPLD) and autosomal dominant polycystic kidney disease (ADPKD). Patients with ADPLD or ADPKD are at risk of developing numerous fluid‐filled cysts in their liver. This leads to hepatomegaly and abdominal distension. It is estimated that approximately 20% of patients will develop symptoms from PLD such as early satiety, reduced mobility (limited forward bending or unable to lie in a prone position), chronic abdominal pain, dyspnea and abdominal wall hernias [1]. PLD is associated with a reduced health‐related quality of life (HRQoL) in particular in those with the most severe phenotype.

The EASL guideline on the management of cystic liver diseases provides a decision‐making aid for the management of different clinical presentations of liver cysts based on their number, location, and size [1]. Singular uncomplicated cysts benefit from targeted aspiration sclerotherapy; locally confined cysts may be treated with surgical fenestration. Patients with severe PLD leading to sarcopenia and other complications may be referred for liver transplantation [1]. However, the difficult‐to‐define intermediate stage, characterised by the presence of smaller, numerous cysts without liver segments spared, remains the most challenging phenotype to manage.

Over the last several years somatostatin analogues (SSAs), such as octreotide, lanreotide and pasireotide have emerged as a therapeutic option for PLD. Cyst growth in PLD is mediated by cyclic adenosine monophosphate (cAMP). Biliary epithelia regulate cAMP‐dependent Cl and HCO_3_ secretion, facilitating fluid accumulation. Secretin, a major cAMP agonist, promotes transporter insertion into cholangiocyte membranes, increasing hepatic cyst fluid secretion. SSAs inhibit cAMP, reducing fluid secretion and cell proliferation. Several clinical trials and meta‐analyses have assessed the efficacy of SSAs in slowing disease progression and improving patient outcomes (Table 1). They collectively highlight the potential benefit of SSAs in managing patients with PLD. SSAs have now been included in the EASL Clinical Practice Guidelines on the management of cystic liver diseases as well as in the KDIGO guideline on ADPKD as a treatment recommendation for symptomatic patients with small to medium‐sized cysts [8, 9]. However, the translation of these data to routine clinical practice comes with challenges. The long‐term benefits and risks of SSAs in this population are unclear while optimal dosing strategies, and patient selection criteria need to be developed.

**TABLE 1:** Characteristics of interventional studies that examined the effect of SSA in PLD.

Author	Year	No. of patients SSA/placebo	Study design	Intervention duration (weeks)	Mean age (years)	Gender, female (%)	Study drug	Liver volume scan	Primary outcomes
van Keimpema [2]	2009	27/27	Randomised, double‐blind, placebo‐controlled trial	24	49.6	97	Lanreotide 120 mg	CT	TLV, HRQoL
Caroli [3]	2010	12/12	Post hoc analysis of a randomised, crossover, placebo‐controlled trial	24	44.5	25	Octreotide LAR 40 mg	CT	TLV
Hogan [4]	2010	29/14	Randomised, double‐blind, placebo‐controlled trial	52	49.7	96	Octreotide LAR 40 mg	MRI/CT	TLV, HRQoL
Pisani [5]	2016	14/13	Post hoc analysis of a randomised controlled trial	156	33.4	63	Octreotide LAR 40 mg	MRI	TLV
van Aerts [6]	2019	93/74	Secondary analysis of a randomised controlled trial	120	49.2	53	Lanreotide 120 mg	MRI	TLV
Hogan [7]	2020	29/12	Randomised, double‐blind, placebo‐controlled trial	52	50.9	93	Pasireotide LAR 60 mg	MRI	TLV, HRQoL

*Note:* Summary of included studies with characteristics such as patient population details, intervention, comparator, and relevant outcomes.

To address this, we assembled a group of multidisciplinary European experts to provide guidance based on a consensus meeting in the framework of an ERN Rare‐Liver workshop. As such the purpose of this position paper is to define the steps in considering SSAs for the management of PLD and to offer a useful and practical guide to physicians involved in the care of these patients.

## 2 Methods

The European Reference Network (ERN) Rare‐Liver facilitated a workshop entitled ‘Harmonisation of somatostatin analogues treatment for Polycystic liver disease’ that took place on April 24–25, 2025 in Hanover/Germany. Participants were selected for their expertise, for diversity in specialty, for geographic region and were provided with comprehensive study information in advance, outlining the objectives of the meeting, and the estimated timing of their contribution.

As a second step, the organisers of the workshop formulated relevant clinical items for discussion such as start criteria, treatment regimen (comparisons of available drugs, dose recommendations, comedication) stop criteria, and outcome measures that come with the use of SSAs in PLD. Those items have been circulated beforehand. Within each area questions were assigned to the panel members based on their individual expertise and the answers were circulated among all the panel for review and discussion over two rounds. A face‐to‐face meeting was held in Hanover to achieve consensus on the recommendations. All group members contributed during this face‐to‐face meeting, using a modified iterative Delphi process. The recommendations are based on the best evidence available at the time of writing and interpreted by expert clinicians involved in the management of these patients. Subjects not meeting the consensus threshold were revised or clarified and re‐evaluated upon further discussion. The process was moderated. For the purposes of this study, consensus was defined as *strong* when agreement reached ≥ 80%, and *weak* when agreement < 80%.

To explore the current clinical practice of the experts concerning the use of SSAs in PLD, we developed a digital questionnaire comprising 15 questions. The questionnaire covered topics such as physician demographics, and indications, choice, dosage and interval of SSA therapy for PLD (Table S1).

## 3 Results

Twelve experts (7 had > 10‐year work experience) present at the workshop completed the questionnaire. Two experts were identified as nephrologists, 10 were gastroenterologists/hepatologists, and all worked in a University Medical Center setting (3 without a liver transplantation program). Eight experts regularly prescribed SSAs for PLD, and 4 started SSAs in 5–10 patients in the last year. Three experts needed prior authorization from the insurance company; there were no barriers for five experts and two experts answered that the prescription of SSA in their healthcare setting was not possible. If prescribed, Lanreotide was the drug of choice (*n* = 7, octreotide, *n* = 1), at a target dosage of 120 mg (*n* = 7) and SSAs were administered in monthly intervals (*n* = 9). The duration of SSA therapy before evaluation of efficacy was variable: < 6 months (*n* = 3), 6–12 months (*n* = 4), > 12 months (*n* = 1). Five experts had a standard‐operating‐procedure/protocol in place for the use of SSA (Table S1).

## 4 Recommendations

**Table:** 

Number	Key question	Recommendation	Level of recommendation
1	Which patients are eligible for SSA treatment?	Patients with Liver volume‐related symptoms in non‐localised disease should be considered for SSA treatment. The assessment of symptoms should be done by disease specific questions, TLV measurement and expected TLV trajectory	Strong
2	Are there contraindications to SSA treatment in polycystic liver disease?	SSA therapy can be considered in symptomatic patients regardless of renal function, although degree of experience from RCTs is limited in advanced CKD (eGFR < 30 mL/min)	Weak
		SSA can be offered to symptomatic patients with history of cyst infection and/or cholelithiasis with careful subsequent monitoring	Weak
3	Which (clinical) parameters are needed to inform the decision to start somatostatin analogue treatment?	Symptomatic burden should be assessed by validated disease specific PROMS (e.g., PLD‐Q or POLCA), TLV (CT/MRI with volumetric analysis) and if available, expected TLV trajectory before treatment start	Strong
4	What are the parameter thresholds to start treatment?	Typical treatment thresholds could include POLCA ≥ 14, PLD‐Q ≥ 32, progression group ≥ 2, or hTLV > 1600 mL/m (KIM ≥ 2)	Weak
5	How to start SSAs?	Treatment should be started at full dose according to clinical trials (Lanreotide 120 mg, Octreotide 40 mg). Dose should be adjusted according to side effects. Patients should be informed about possible side effects	Strong
6	What are the goals (outcome measures/endpoints) of SSA treatment for polycystic liver disease?	The ultimate goal of SSA treatment in PLD is the relief of symptoms related to liver volume	Strong
7	What are the parameters to assess response on follow‐up?	PROMS and TLV measurements can be continued at follow‐up to document further stabilisation of the disease	Strong
8	How do we define response?	Symptom relief without hTLV progression is indicative of treatment response within the first 6–12 months (e.g., PROM at least after 6 months, hTLV measurements at least after 12 months)	Strong
9	How long do we need to treat patients with SSA?	SSA may be continued (as long as benefits overweight side effects) until an attenuation of disease progression by natural disease course is expected	Strong
10	What are the thresholds of the parameters to stop treatment?	Stop when the treatment response criteria are no longer being achieved	Strong
11	Which adverse events are common and how do we manage them?	Patients should be aware of side effects before starting treatment and informed that gastrointestinal abnormalities, especially steatorrhea, are common side effects, that can be ameliorated by avoiding fatty food and will disappear spontaneously later on	Weak

1. **Which patients are eligible for SSA treatment?**

**Patients with liver volume‐related symptoms who have non‐localised disease should be considered for treatment. The assessment of symptoms should be performed by disease‐specific questions, TLV measurement and expected TLV trajectory**

**FIGURE 1:**
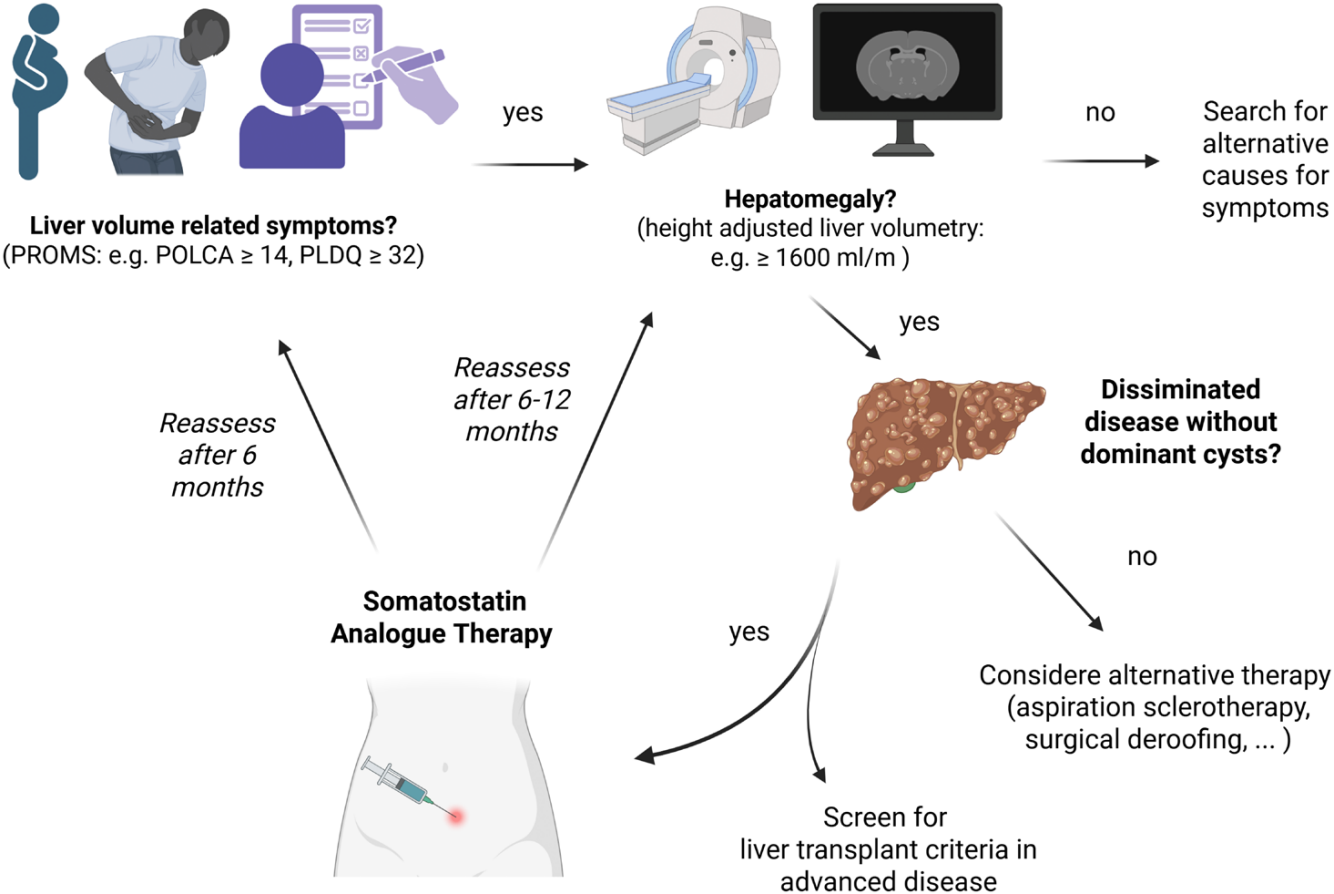
Flowchart for decision‐making for medical therapy in severe PLD.

The primary goal of PLD treatment is symptom relief. It is key to establish a relation between the liver phenotype and symptoms in patients with PLD (Figure 1). Here, liver volumetry (additional kidney volumetry in patients with ADPKD) and the use of validated PROMs should be used to assess the current disease stage. If a center does not offer volumetric assessment, patients should be referred to specialised centers before starting therapy. Patients with PLD who are asymptomatic should not be offered treatment. Given the non‐specific nature of abdominal symptoms, a broader differential diagnosis is essential, particularly in PLD patients with minimal liver enlargement. In these patients a search for an alternative explanation of the (abdominal) symptoms should be pursued. The current thinking is that hepatomegaly caused by multiple cysts is the main reason for PLD‐specific symptoms such as abdominal distension, abdominal pain in the rib cage, sides, abdomen or back, early satiety, limited mobility and shortness of breath. In symptomatic patients, liver volumetry by CT or MRI should be pursued. A large number of interventional trials (Table 1) have utilised these imaging modalities to quantify total liver volume (TLV) as an endpoint. Currently, a standardised method for liver volumetry is lacking, and we await the advent of automated volumetry of PLD in routine clinical practice. However, longitudinal assessments contribute to a better understanding of the natural history of the disease for the individual patient. Therefore, serial imaging, using the same modality, yields important data that facilitates clinical decision‐making. The genetic background of PLD, ADPKD or ADPLD, is not of relevance for the decision to start SSA therapy, because both diseases exhibit similar treatment response rates [2].

2 **Are there contraindications to SSA treatment in polycystic liver disease?**

**SSA therapy can be considered in symptomatic patients regardless of kidney function, although evidence from RCTs is limited in advanced CKD**

**SSA can be offered to symptomatic patients with a history of cyst infection and/or cholelithiasis with careful subsequent monitoring**

**SSAs should not be offered during pregnancy or while breastfeeding**.

A reduction of the Lanreotide dose has been suggested in patients with impaired renal function. A pharmacokinetic study in haemodialysis patients demonstrated reduced serum clearance and volume of distribution, supporting dose reduction in severe renal impairment [10]. Although Lanreotide has a broad therapeutic index with limited correlation between serum levels and adverse effects, a recent randomised clinical trial (RCT) in ADPKD patients with declining eGFR implemented dose adjustments based on renal function [11]. Patients with eGFR ≥ 30 mL/min received 120 mg, while those with eGFR < 30 mL/min received 90 mg. Another RCT examined octreotide (40 mg LAR) in a high‐risk population of ADPKD patients (eGFR 15–40 mL/min) and specifically documented safety and efficacy regarding a renal endpoint [12].

Studies have documented occurrences of cyst infections with SSA use, but it is unclear if and to what extent SSA or other factors contribute to the risk. A large clinical trial evaluating lanreotide (120 mg/28 days) in 309 patients with ADPKD (eGFR of 30–60 mL/min/1.73 m^2^) reported a higher incidence of cyst infections with lanreotide (*n* = 7) compared to placebo (*n* = 0). A history of a cyst infection was the main risk factor for these episodes in these patients. From the literature, it is clear that renal transplantation with concomitant immunosuppressants also constitutes a major risk factor for cyst infections [13]. Therefore, patients with a history of cyst infection and/or patients with ADPKD and a kidney transplant may not be ideal candidates for SSA therapy [6, 13].

SSA therapy during pregnancy and breastfeeding is controversial due to limited data on SSA therapy and pregnancy. However, there are individual case reports of pregnant patients with endocrine tumours undergoing SSA therapy that have shown a favourable outcome for both mother and child [14].

3 **Which (clinical) parameters are needed to inform the decision to start somatostatin analogue treatment?**

***Symptomatic burden should be assessed by validated disease‐specific PROMS (*e.g., *PLD‐Q or POLCA), TLV (CT/MRI with volumetric analysis) and if available, expected TLV trajectory before treatment start***

Two disease‐specific symptom severity questionnaires, PLD‐Q (Polycystic Liver Disease Questionnaire) and POLCA (polycystic liver disease complaint‐specific assessment) have been developed to monitor disease activity in PLD [15, 16]. The PLD‐Q serves as a disease‐specific questionnaire and is based on reporting (with a weekly recall) of 16 items each consisting of one question in which the patient indicates the severity or frequency of a specific symptom and a follow‐up question assessing the impact on daily life. Questions focus on items such as ‘feeling full’, ‘lack of appetite’, ‘early satiety’, ‘nausea’, ‘pressure or pain rib cage’, and ‘limited mobility’. PLD‐Q has been validated both in European and American populations [15]. POLCA captures the presence and severity of nine hepatomegaly‐related symptoms divided into four subscales: perception of enlarged liver volume, impact of food intake, gastro‐oesophageal reflux disease, related complaints and severity of perceived illness. POLCA was validated in a Belgian population [16, 17]. PLD‐Q has served to evaluate the effect of aspiration sclerotherapy, and that of cyst fenestration as well as resection procedures. POLCA was developed to guide decision‐making for liver transplantation assessment and to evaluate the effect of SSA therapy. Both PLD‐Q and POLCA are specifically equipped to identify symptomatic patients with PLD and quantify PLD‐related symptoms.

The future TLV trajectory of an individual may be predicted using the PLD‐Progression Grouper using a single liver volumetry measurement [18, 19]. This is an interactive web application designed to assist in the prognostic assessment of PLD. It models disease progression by integrating patient age and TLV to estimate the annual percentage growth rate of liver volume and categorises individuals into three distinct risk groups based on progression severity. This application operates on the principle that the standard baseline TLV at age 20 years is 850 mL/m and increases linearly. In clinical practice it allows identification of patients who may benefit from early intervention and may help to prioritise patients for liver transplant evaluation.

4 **What are the thresholds of the parameters to start treatment?**

**Typical treatment thresholds could include POLCA ≥ 14, PLD‐Q ≥ 32, progression group ≥ 2, or hTLV > 1600 mL/m (KIM ≥ 2)**

A set of questions specific to PLD should be asked to ascertain liver volume related symptoms (LVrS) (abdominal distension, abdominal pain in the rib cage, sides, abdomen or back, early satiety, limited mobility, difficulties in bending over, cutting toenails and shortness of breath, e.g.). Ideally, these questions should be asked in the context of a structured and validated questionnaire like PLD‐Q and POLCA as described above. PLD‐Q scores range from 0 to 100 and a threshold of ≥ 32 points identifies patients who might benefit from treatment. For POLCA the thresholds to consider treatment with SSAs or liver transplantation are set at an SPI ≥ 14 and at an SPI ≥ 18, respectively. However, so far there is no robust cut‐off level, that predicts response to SSA therapy in both scoring systems. From KIM stage 2 onward (a moderate PLD with a height‐adjusted total liver volume (hTLV) > 1600 mL/m) and in patients classified as progression group 2 (indicating an intermediate rate of liver volume increase and a higher risk of symptomatic disease progression), treatment with SSA should be considered. KIM 2 reflects the volumetric disease stage, while progression group 2 reflects the dynamic component of disease growth over time [19]. Even in the absence of a high symptom burden as reflected by POLCA or PLD‐Q scores, SSA therapy should be considered when rapid cyst growth is confidently predicted, given its potential to limit disease progression.

5 **How to start SSAs?**

**Treatment should be started at full dose according to clinical trials (Lanreotide 120 mg, Octreotide 40 mg). Dose should be adjusted according to side effects. Patients should be informed about possible side effects**.

RCTs have usually utilised a single, full‐dose regimen. The most robust dataset in terms of patient numbers and follow‐up duration comes from the DIPAK1 study with Lanreotide, with over 200 patients followed for up to 29 months. However, full‐dose Lanreotide was tolerated by only a minority of patients (34%) [6], whereas Octreotide may provide a more favourable tolerability profile, with > 80% of patients tolerating the full dose [20]. Classical dose‐finding studies are lacking; however, subgroup analyses suggest a dose–response relationship favouring higher efficacy at increased doses (Table 1) [21]. We do not recommend dose escalation beyond those used in RCTs. Of note, an ongoing Phase 2 trial (POSITANO; NCT05281328) is comparing two different dosing regimens (CAM2029 10 mg once weekly or every two weeks) of a novel Octreotide formulation which may shed some light on the issue. To minimise injection‐site reactions, alternating injection sites are recommended. Patients should receive training prior to self‐administration.

While all three available SSAs (Octreotide, Lanreotide, Pasireotide) demonstrated comparable efficacy in RCTs, Pasireotide has been associated with a higher incidence of adverse effects, notably impaired glucose tolerance. Therefore, Pasireotide is not recommended as first‐line therapy. To mitigate gastrointestinal adverse effects—largely attributable to transient exocrine pancreatic insufficiency observed in up to 70% of patients in early trials [2]—patients should be advised to avoid high‐fat meals in the peri‐injection period. These symptoms typically resolve with continued therapy and do not generally necessitate treatment discontinuation. Dose reduction was effective in alleviating gastrointestinal symptoms in 86% of affected patients [6]. In cases of persistent symptoms, pancreatic enzyme replacement therapy may be considered.

6 **What are the goals (outcome measures/endpoints) of SSA treatment for polycystic liver disease?**

**The ultimate goal of SSA treatment in PLD is the relief of symptoms related to liver volume**

Patients with PLD may experience severe impairment in all aspects of their quality of life due to liver volume‐related mass effects. Available evidence suggests that both TLV and growth rate are responsible for the disease burden and should be considered before the start of therapy [2, 5, 18, 22]. TLV should be quantified via CT/MRI image‐based segmentation and if available, calculation of the expected TLV trajectory before treatment start (Figure 1) as discussed in question 3.

A reduction of TLV per se is not the primary treatment goal, but TLV serves as a measure of the plausibility that the symptoms are indeed provoked by the hepatomegaly and an assessment of treatment response (see also questions 7. How do we define response and 8. What are the parameters to assess response on follow‐up). A preemptive therapy of PLD hepatomegaly in asymptomatic patients is not justified since the majority of PLD patients will remain asymptomatic and there is no expected negative outcome of a treatment initiation after the appearance of symptoms [8]. Untreated symptomatic PLD patients score significantly worse on HRQoL measures such as EuroQol‐visual analogue scales (EQ‐VAS) and Short Form 36 (SF‐36) or Physical Component Summary (PCS) score compared to age and gender‐matched populations. However, SSA therapy in PLD does not consistently improve these generic HRQoL measures, in most part because of the uneven adverse event profile of the various SSA. In a meta‐analysis the potential benefits of Lanreotide and Octreotide were obscured by the adverse event profile of Pasireotide [23], whereas individual RCTs have demonstrated that TLV reduction is associated with improvement of measures of generic HRQoL scales in the majority of patients with PLD (Ref. Table 1). Disease‐specific questionnaires (e.g., PLD‐Q or POLCA) are better suited to capture disease activity [24]. Somatic symptoms in PLD are associated with stage of hepatomegaly and can be effectively captured through these PLD‐specific PROMs. Use of these PROMS should be regarded as a relevant outcome measure for treatment initiation, evaluation and continuation of SSA in PLD [25].

There is currently no robust evidence suggesting that SSA mitigates progression to liver‐related endpoints in PLD.

7 **What are the parameters to assess response on follow‐up**

**PROMS and TLV measurements can be continued at follow‐up to document further stabilisation of the disease**

**FIGURE 2:**
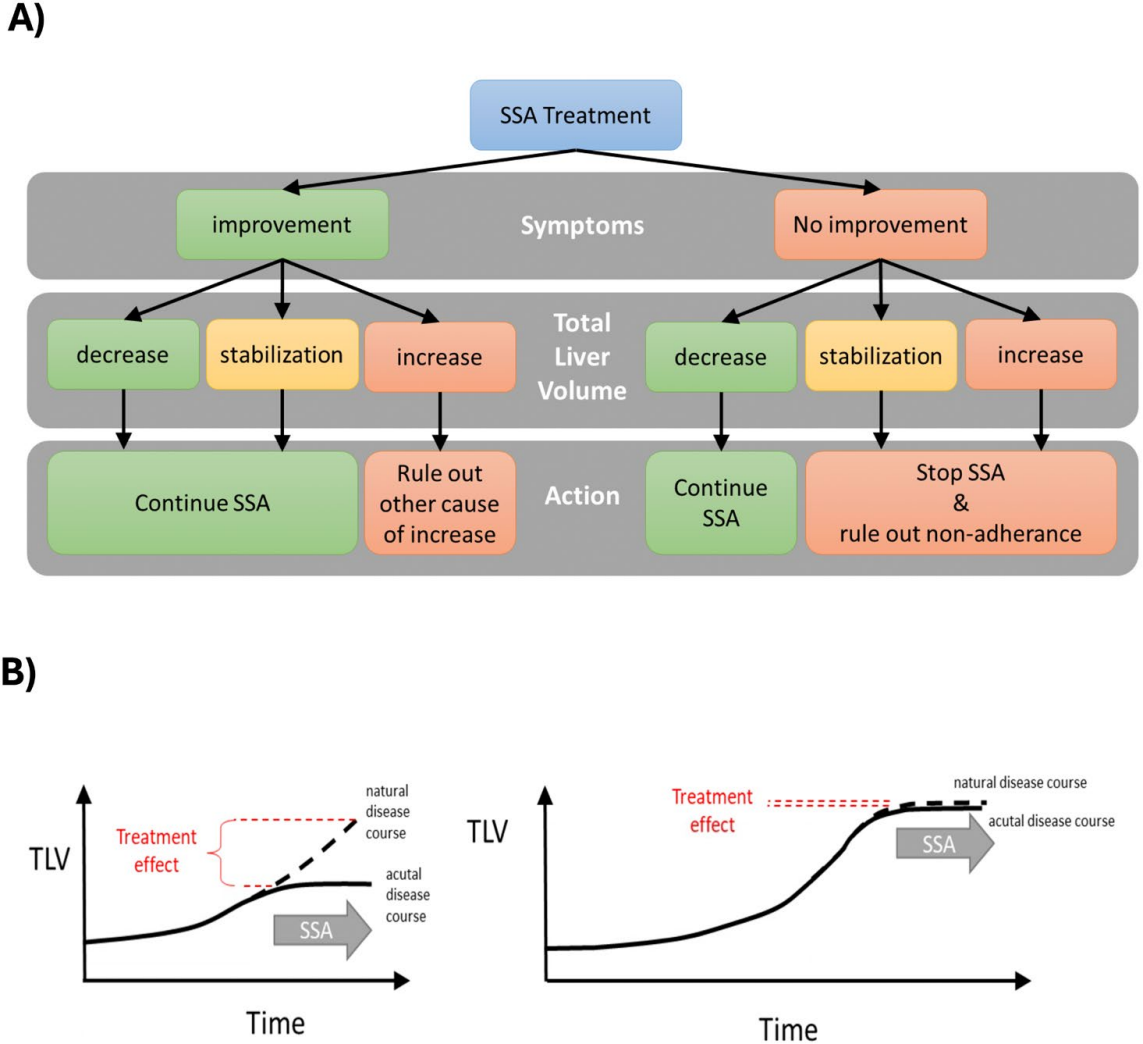
Evaluation of treatment response of SSA therapy in severe PLD. (A) Flow chart on the translation of longitudinal changes of liver volume and volume related symptoms into clinical decisions making regarding medical therapy. (B) Definition of treatment response despite no improvement of liver volume via the comparison of the expected natural and actual disease course under SSA therapy. Stabilisation of liver volume can be considered as a positive treatment response in disease periods with expected liver growth (left panel) but as insufficient response in periods of naturally stable liver volume (right panel).

In clinical practice, global evaluations of the perceived impact of therapy (Figures 1 and 2) may be used in conjunction with PROMS. To ensure optimal comparability, follow‐up assessments should employ the same validated instruments—such as the POLCA and PLDQ questionnaires—used prior to the initiation of SSA therapy.

While the symptomatic response remains central, the volumetric change in TLV constitutes a secondary, yet critical, follow‐up biomarker to estimate SSA efficacy. Consistency in imaging methodology and volumetric analysis before and during treatment is essential for accurate longitudinal assessment.

The reassessment of SSA therapy should also include the screening for relevant adverse events associated with SSA such as gastrointestinal complaints, glucose intolerance, formation of gallstones and cyst infections.

8 **How do we define response?**

**Symptom relief without hTLV progression is indicative of treatment response within the first 6–12 months (e.g., PROM at least after 6 months, hTLV measurements at least after 12 months)**

***PROMS and TLV measurements can be continued at follow‐up to document further stabilisation of the disease***

The primary treatment goal of SSA‐based treatment is to improve symptoms and the current evidence suggests that both TLV at a given time and growth rate, are main contributors to the severity of the disease. Accordingly, TLV should be incorporated into the decision‐making process when selecting an appropriate therapeutic strategy. A reduction in TLV remains the most direct and intuitive indicator of a favourable treatment response. Evidence from RCTs indicates that Lanreotide therapy leads to a reduction in TLV in up to 60% of patients but a similar effect was seen in approximately 30% of patients in the placebo group also, highlighting the need for careful interpretation of treatment effects [6]. Not only a decrease but also stabilisation of TLV, in conjunction with relief of liver volume‐related symptoms, may be regarded as a valid indicator of therapeutic response. Notably, TLV stabilisation during periods typically characterised by TLV growth is a recognised effect of SSA therapy, which can positively influence the trajectory of liver volume expansion (Figure 2). This trajectory may be inferred from serial liver volumetric assessments or by categorising patients into PLD progression groups, depending on the availability of multiple or single pretreatment liver volumetries [18]. However, there are no robust cut‐offs in liver volume increase or decrease, that can be applied. There is interindividual variability in the course of disease progression among patients. Therefore, a fixed cut‐off should not be the primary goal; rather, the individual patient's delta, in combination with other parameters, should guide clinical decision‐making. The same applies for PLD‐Q and POLCA. There are no validated specific cut‐offs with regard to SSA therapy. However, the increase or decrease of the results allows a more objective evaluation of the clinical response to therapy of the patient.

Conversely, if symptomatic relief is not accompanied by stabilisation or reduction in TLV, further evaluation is warranted to identify alternative etiologies of abdominal symptoms or additional contributors to liver growth, such as exogenous oestrogen exposure (Figure 2). In cases where TLV does not decrease and therapy adherence is confirmed, a lack of improvement in liver‐related symptoms should be interpreted as an absence of therapeutic response, and SSA should be terminated.

9 **How long do we need to treat patients with SSA?**

**SSA may be continued (as long as benefits outweigh side effects) until an attenuation of disease progression by natural disease course is expected**

Although a drop in TLV can occur immediately in the first three months, the minimum treatment duration in the available RCTs was 6–12 months (Table 1) [2, 3, 4]. Therefore, we suggest evaluating treatment response not earlier than after 6–12 months. Data on treatment response is available for up to 3 years in some of the clinical RCTs (see Table 1). Clinical experience also supports the fact that long‐term improvements can be maintained with treatment of SSAs beyond 3 years with further decrease of TLV in the second year of treatment [20].

Early discontinuation can lead to rebound in both liver volume and disease‐related symptom burden within 3–4 months after withdrawal. However, SSA therapy can be reintroduced with good treatment response after a drug holiday [6, 20, 26]. It should be noted that a recurrent increase in liver volume after cessation complicates further treatment [27]. Therefore, SSA therapy may be continued until tolerability becomes an issue or the expected course of disease evolution suggests that treatment may be stopped (e.g., in a female who has lived through the menopause).

10 **What are the thresholds of the parameters to stop treatment?**

**Stop when the treatment response criteria are not achieved any more**.

Evaluation of hepatic volume response to SSA treatment should be deferred until a minimum of six months has elapsed, as premature assessments may not reflect the true trajectory of therapeutic effects as discussed above. Acute and significant decreases in liver volume, particularly when abrupt, should raise suspicion of cyst rupture, which represents an incidental event rather than a beneficial outcome of treatment. Conversely, in patients—most commonly younger females—who experience a rapid increase in liver volume despite ongoing therapy, the medical regimen may serve primarily as a bridging prior to liver transplantation. In such cases, discontinuation of therapy is strongly discouraged, particularly in patients already listed for transplantation, as this may precipitate a rebound of liver growth and as a consequence, exacerbate symptoms (Figure 2).

Among postmenopausal women, continued therapy may be considered for an extended duration (3–5 years), after which a probatory cessation may be attempted. If no rebound effect is observed, treatment discontinuation may be definitive. However, further volume reduction may be observed with SSA beyond the initial 3–5 year window, suggesting potential benefit from continued administration [20]. Patients should be counselled that treatment cessation does not curtail the efficacy of future retreatment as there is data suggesting that the response is similar on retreatment after interruption of SSA treatment. Early termination of therapy, particularly without appropriate planning, may result in increased patient distress and should therefore be handled with caution.

11 **Which adverse events are common and how do we manage them?**

**Patients should be aware of side effects before starting treatment and informed that gastrointestinal complaints, especially steatorrhea, are common side effects, that can be ameliorated by avoiding fatty food and will disappear spontaneously later on**.

The label for Octreotide and Lanreotide mentions four categories of warnings and precautions for SSAs: (1) Cholelithiasis (2), hypoglycemia or hyperglycemia [5, 14], (3) hypothyroidism, and (4) bradycardia or cardiac arrhythmia.

Gastrointestinal symptoms are among the most frequently reported adverse events with SSAs [6, 12, 13]. These include steatorrhea and abdominal pain, which are typically mild to moderate and transient. In clinical studies, gastrointestinal‐related adverse events occurred in approximately 67%–94% of patients. Management strategies involve patient education, dietary modifications (avoidance of fatty food intake in the days following the injection), and the on‐demand use of pancreatic exocrine replacement therapy, particularly around the first 2–3 SSA injections [28].

Physicians should be aware that possible, less common side effects of SSA include cholelithiasis and biliary complications [17]. The best documented evidence comes from a RCT that investigated Lanreotide in patients with ADPKD. Over a 2.3‐year treatment period with lanreotide, 15% of patients (*n* = 124) developed new gallstones, compared to 1% in the untreated group (*n* = 125). Gallstones were typically multiple (> 20) and small (≤ 3 mm). 9/19 patients with treatment incident gallstones developed biliary complications, often after discontinuation of lanreotide. These findings suggest that asymptomatic gallstones present at baseline do not confer a high risk of complications, unlike incident gallstones that develop during therapy. Thus, the presence of pre‐existing asymptomatic gallstones should not be considered a contraindication to initiating SSAs. Monitoring and evaluation of the presence of choledocholithiasis after discontinuation of SAAs may be warranted, since complications due to gallstones more often occur after discontinuation of than under therapy [29]. SSAs may cause alterations in glucose metabolism, potentially leading to both hyperglycemia and hypoglycemia. Regular monitoring of blood glucose levels is recommended, particularly in diabetic patients or those at risk for metabolic disorders. Adjustments in antidiabetic medication may be required to maintain optimal glycemic control.

SSAs may cause suppression of TSH through a direct suppression of somatostatin receptors in the pituitary gland. This may lead to mildly lower TSH levels. TSH suppression usually does not change thyroid hormone levels and is reversible upon discontinuation of SSA. Thyroid function monitoring may be advised in patients with pre‐existing thyroid disease.

Conduction abnormalities have been observed in patients with acromegaly as part of their phenotype, unlike in patients with PLD. In patients with acromegaly injections with SSA may lead to bradycardia and clinical vigilance is advisable in other populations [30].

Pain and the development of nodules at the injection site are among the commonest adverse events with SSA (e.g., up to 50%) [2]. While specific frequencies vary, these reactions are generally mild and resolve spontaneously. Proper injection techniques, including rotating injection sites and administering the medication deeply into the subcutaneous tissue, can help minimise discomfort.

Hair loss may occur with prolonged treatment, is of significant concern for patients and is an often‐cited factor for early cessation of therapy.

Dose reductions of SSAs (e.g., lanreotide to 90 mg or 60 mg) may be required in approximately 10% of patients to manage adverse events. While this helps to improve the safety profile, it also compromises efficacy. Individualised titration optimises therapeutic efficacy while minimising treatment‐limiting toxicity [6].

An annual non‐contrast low‐dose CT scan yields about 2–5 mSv per exam. Over 20 years, this cumulative dose may slightly increase lifetime cancer risk by only a few tenths of a percent, depending on age, sex, and susceptibility [31, 32]. The clinical benefit should always be weighed against this potential long‐term risk. However, the usual course of the disease does not require annual CT scans over a long lifetime period. To further address this issue, MRIs can be preferred.

## 5 Future Research Agenda

There is an overwhelming body of robust evidence that favours the beneficial treatment effect of SSA on TLV and liver volume related symptoms (LVrS). However, the translation of these RCT results into routine clinical practice is limited by the heterogeneity of the trials (Table 1).

The ERN Rare Liver workshop identified a number of unanswered areas for research:

- ***Map the natural history of PLD and identify aggressive sub phenotypes:*** The natural disease course with the trajectories of LVrS and TLV can still be predicted insufficiently, and current progression models need further validation [18]. This still leads to a trial‐and‐error approach of SSA therapy.
- ***Better understanding of the interplay between kidney and liver volume in ADPKD***: It is clinically challenging to understand the origin of volume‐related symptoms (kidney and/or liver) in ADPKD patients. A better understanding of the interplay and better prediction tools of trajectories of organ volume and organ function would help to anticipate the patients' medical need.
- ***Efficacy of preemptive treatment***: The current approach is to wait for the onset of LVrS and relevant hepatomegaly before volume‐reducing therapies are offered, because the majority of patients remain asymptomatic. A better understanding and prediction of the disease course would help to select those patients with the highest medical need. Preemptive therapy approaches might be tested in these patients ‘at risk’ to move from the treatment of symptoms to prevention.
- ***Efficacy of SSA*** The median treatment effects of SSA, as helpful as they are for symptomatic patients, with reduction rates of 2%–8% of TLV do not completely change the trajectory of the disease. Identification of new molecules and the further exploration of the benefits of ursodeoxycholic acid remain unmet needs.
- ***Efficacy of alternative modalities*** Exploration of selective radiological modalities to curtail liver cyst growth such as hepatic artery embolization or selective internal radiation therapy (SIRT).

## 6 Discussion

Severe PLD is a rare phenotypical expression of ADPLD and ADPKD and the use of SSA to curtail its symptoms has emerged as a management option for these patients. This position paper provides a comprehensive overview of the role of SSA in severe PLD and is based on a consensus‐driven process involving a multidisciplinary panel of experts. We addressed aspects such as patient selection, biomarkers and outcome measures, start and stop criteria, management of adverse events, and contraindications. This led to 11 actionable recommendations that help to navigate the key decision points of SSA therapy in severe PLD.

SSA appears to be the only medical option in PLD that has withstood rigorous testing in RCTs. All RCTs executed to date have documented a statistically significant reduction of total liver volume regardless of SSA used, favouring a class effect. However, the safety profile differs. Individual patient analysis shows that approximately 60% of patients respond with a decrease in liver volume, a result independent of baseline factors, except age and gender [6]. There is also a meaningful reduction in the polycystic kidney volume with treatment. However, this does not translate into an improved trajectory of the renal function decline, which restricts the indication of SSA in ADPKD to the liver manifestations.

We identified several barriers to the use of SSA in clinical practice. TLV is used as a primary endpoint in all RCTs and requires volume measurement. Manual segmentation of polycystic liver is reliable, but time‐consuming. Several companies have developed semi‐ and fully automatic segmentation programs to measure liver volume, but access to these tools in routine clinical practice is limited. The PROMS PLD‐Q, and POLCA may serve as alternative biomarkers, but both questionnaires have not been tested in a randomised SSA‐based trial. Although regarded as safe drugs, SSA has safety issues. This position paper provides recommendations on how to manage potential adverse events so that prolonged exposure may be allowed.

Reimbursement remains a challenge across Europe. In some countries, SSA therapy is covered without the need for a separate application, while in others, physicians may submit an individual request to the respective health insurance provider for cost approval. However, in several countries, no reimbursement option exists at all, making prescription for patients nearly impossible. All of the SSAs developed so far are off‐label for PLD. CAM2029, a novel long‐acting subcutaneous depot formulation of octreotide has been granted orphan drug designation by European and U.S. regulators for the treatment of PLD. A Phase 2/3 randomised, double‐blind, placebo‐controlled trial in 71 patients with PLD has just been completed and trial results are awaited. If results are favourable, a labelled treatment option could be available in the near future.

In conclusion, this consensus statement sheds light on critical aspects of SSA‐based therapy for PLD. The primary goal is to reduce liver‐related symptoms by stabilising or decreasing liver volume. Treatment response is defined by symptom relief and absence of hTLV progression over 6–12 months. Response is monitored using PROMS (e.g., PLD‐Q, POLCA) and volumetric imaging. Treatment should begin at full dose and continue as long as benefits outweigh side effects. Indications include non‐localised, symptomatic PLD with thresholds like PLD‐Q ≥ 32 or hTLV ≥ 1600 mL/m. Common side effects include gastrointestinal issues, while advanced chronic kidney disease or cyst infections require cautious use.

## Author Contributions

All authors contributed to the conception and design of the study. R.T. and J.P.H.D. coordinated the Delphi process, including expert recruitment, survey development, and data collection. S.H. performed data analysis and synthesised the findings. S.H., F.N., R.T., J.P.H.D. provided methodological expertise and contributed to the interpretation of the results. S.H., R.T. and J.P.H.D. drafted the initial manuscript, with substantial input from all authors. All authors critically revised the manuscript for important intellectual content and approved the final version for publication.

## Conflicts of Interest

The authors declare no conflicts of interest.

## Supporting information


**Table S1:** liv70451‐sup‐0001‐TableS1.docx.

## Data Availability

No new data were generated or analyzed for this work; all data referenced are from previously published studies.
