# Supplementary material for: Medical Management of Polycystic Liver Disease: A Position Statement From the European Reference Network on Hepatological Diseases
Source: Liver Int. 2025 Dec 2;46(1):e70451. doi: 10.1111/liv.70451 (PMC12670357; doi:10.1111/liv.70451)
Supplement: Supplementary file 1 — Table S1: liv70451‐sup‐0001‐TableS1.docx. [file LIV-46-0-s001.docx]

Supp. Table 1

| **Experts participating at the ERN meeting April 24^th^/25^th^ in Hanover ^30^** | **N** |
| --- | --- |
| **Profession (N)**   - Nephrologist - Hepatologist/ Gastroenterologist | 2  10 |
| **Time as a consultant**  >10 years  5-10 years  < 5 years | 7  4  0 |
| **Working Place**  University Hospital with transplantation program  University Hospital without transplantation program  General Hospital | 8  3  0 |
| **Volume reducing therapy available at the center**  Aspiration sclerotherapy  Surgery | 7  11 |
| **Number of PLD patient under professionals' care**  > 10 patients  5-10 patients  <5 patients | 11  0  0 |
| **Ability to prescribe SSAs for PLD patients in the health care system**  Yes  No | 4  8 |
| **Number of SSAs prescriptions (patients) within the last year**  >10  5-10  1-5  0 | 0  4  4  0 |
| **Prescribed Drug**  Pasireotide  Octreotide  Lanreotide | 0  1  7 |
| **Dosage interval**  Monthly  weekly | 8  0 |
| **Dosage prescribed in mg**  Lanreotide (120)  Octreotide (40)  Depending on tolerance | 7  1  2 |
| **Average treatment duration before reevaluation**  Individual decision  >12 months  6-12 months  <6 months | 2  1  4  3 |
| **Examination performed before starting treatment**  Ultrasound  CT/MRI  Exclusion of other causes for symptoms (e.g. endoscopy) | 1  8  2 |
| **Barriers for prescription**  Reimbursement is not available  Prior authorization of insurance needed  No barriers | 2  3  5 |
| **Assessment of response**  PROMs  CT volumetry  I ask them | 6  8  7 |
| **SOP available at the Center**  Yes  No | 5  6 |
